# Pediatric Pneumococcal Serotypes in 4 European Countries

**DOI:** 10.3201/eid1609.100102

**Published:** 2010-09

**Authors:** Germaine Hanquet, Esther Kissling, Asuncion Fenoll, Robert George, Agnes Lepoutre, Tinne Lernout, David Tarragó, Emmanuelle Varon, Jan Verhaegen

**Affiliations:** Author affiliations: Scientific Institute of Public Health, Brussels, Belgium (G. Hanquet, E. Kissling, T. Lernout);; Belgian Knowledge Centre, Brussels (G. Hanquet); Instituto de Salud Carlos III, Madrid, Spain (A. Fenoll, D. Tarragó,);; Health Protection Agency, London, UK (R. George);; Institut de Veille Sanitaire, Saint Maurice, France (A. Lepoutre);; Hôpital Européen G. Pompidou, Paris, France (E. Varon);; Katholieke Universiteit Leuven Pneumococcus Reference Laboratory, Leuven, Belgium (J. Verhaegen)

**Keywords:** Invasive pneumococcal disease, pneumococcal conjugate vaccines, serotype, bacteria, France, Spain, Belgium, England, Wales, research

## Abstract

After heptavalent pneumococcal conjugate vaccine (PCV7) was marketed in France, Spain, Belgium, and England and Wales (United Kingdom), invasive disease from non-PCV7 serotypes (NVT) increased. Adjusted serotype-specific incidences among children <15 years of age were compared between 1999–2002 (prevaccine) and 2005–2006 (postmarketing). Vaccine coverage increased to ≈32%–48% in France, Spain, and Belgium but remained <1% in England and Wales. Serotype 1 incidence rose in all age groups and countries (incidence rate ratio [IRR] 1.3–4.2; p<0.004), independently of PCV7 use, but incidence of serotypes 7F and 19A increased most in France, Spain, and Belgium (IRR 1.9–16.9 in children <5 years; p<0.001), where PCV7 coverage was greater. Vaccine-induced replacement of PCV7 serotypes possibly contributed to NVT increases, as did secular trends. New vaccines targeting these serotypes are available, but serotype dynamics needs further exploration that accounts for underreporting and prevaccine trends.

## Abstract 2

TOC Summary: Non–heptavalent pneumococcal conjugate vaccine serotypes have increased in Spain, France, Belgium, and England and Wales.

*Streptococcus pneumoniae* is a leading cause of meningitis and septicemia worldwide. More than 90 serotypes have been identified for *S. pneumoniae*, but serotype distribution differs by area and changes over time (*1**–**3*). The heptavalent pneumococcal conjugate vaccine (PCV7) targets the 7 serotypes—4, 6B, 9V, 14, 18C, 19F, and 23F—that most commonly caused pediatric invasive pneumococcal disease (IPD) in the United States. Widespread PCV7 use in the United States since 2000 led to rapid and dramatic decreases in vaccine serotypes and an overall decrease of IPD incidence (*4*).

In Europe, PCV7 was licensed for pediatric use in 2001 and marketed in Spain and France in 2001, England and Wales in 2002, and Belgium in 2004; the 7 serotypes accounted for 68%**–**77% of IPD cases in children <2 or <5 years of age (*5**–**8*). Vaccination policies from marketing to introduction of PCV7 into the universal vaccination schedule differed among countries. In Spain, Belgium, and France, vaccination aimed to reach a progressively increasing proportion of children <2 years of age, resulting in low but increasing vaccine coverage (36%**–**50% of young children in 2005–2006) because PCV7 was not free for all of them (*6**,**7*). In England and Wales, until late 2006 PCV7 was recommended only for medical risk groups, and population-level vaccine coverage was negligible. PCV7 was introduced in the national universal program and delivered free in France and England and Wales in 2006 and Belgium in 2007. In Spain, free universal vaccination was limited to the Madrid region beginning in 2006.

Several PCV7 postlicensure studies in the United States and European countries have described substantial increases in non-PCV7 vaccine serotypes (NVTs) (*6**,**7**,**10**–**13*). These findings raised concern that vaccine use could lead to replacement of PCV7 serotypes by NVTs, as occurred with pneumococcal carriage (*14*).

In Spain, Belgium, France, and England and Wales, NVT disease increased substantially between marketing and introduction of PCV7 into the universal schedule, when vaccine use was moderate in Spain, Belgium, and France and negligible in England and Wales. Considering that a 10-valent vaccine and a 13-valent conjugate vaccine are licensed in the European Union (EU), a better understanding of the dynamics of the additional serotypes is needed to help decision making on future vaccine strategies. This study describes and compares temporal trends of PCV7 serotypes and NVTs among children in 4 European countries, taking into account the levels of PCV7 use. We focused on the emergence of serotypes 1, 7F, and 19A because they were responsible for most of the NVT increase.

## Methods

This population-based study is based on surveillance data collected prospectively by the national reference laboratories (NRLs) of Spain, Belgium, France, and England and Wales during July 1996–June 2006. (In the United Kingdom, Scotland and Northern Ireland were not included in this study because they use separate surveillance systems.) IPD isolates were referred by laboratories throughout each country to the NRL. IPD surveillance among children was enhanced in England and Wales, France, and Belgium starting in 1996, 2002, and 2005, respectively, by encouraging microbiologists to systematically refer pneumococcal isolates to the NRL for typing.

### Definitions and Inclusion Criteria

We included all IPD cases, defined by isolation of *S. pneumoniae* from a normally sterile fluid in children <15 years of age and referred to the NRL of their country. One isolate per disease episode was used in the analysis. Serotypes targeted by PCV7 were grouped as vaccine types (PCV7 types). All other serotypes were considered NVTs. Meningitis was defined as isolation of *S. pneumoniae* in cerebrospinal fluid (CSF).

An epidemiologic year was July through June. A prevaccine period was defined as the 3 epidemiologic years during July 1999–June 2002. The postmarketing period was 2005–2006.

### Microbiologic Testing

Serotype and antimicrobial susceptibility were determined by each NRL as described (*9**,**15**–**17*). In Spain, all strains of serogroups 6 and 19 were subjected to PCR serotype identification (*18*). In Belgium during 1996**–**2004 (before PCV7 marketing), 36% of isolates had the serogroup but not the serotype determined (17% for serogroup 19). However, all serogroup 19 isolates from children <2 years of age were typed, and during the postmarketing period, the serotype was determined for all isolates received. Isolates with missing serotype were assumed to follow the same serotype distribution as isolates from the same serogroup, by year and patient age group.

Isolates were considered susceptible, intermediate, or resistant to antimicrobial drugs according to Clinical and Laboratory Standards Institute criteria (penicillin intermediate and resistant, MIC 0.12**–**1.0 mg/L and MIC >1 mg/L, respectively; erythromycin resistant, MIC >0.5 mg/L) (*19*). England and Wales data on antimicrobial drug resistance were not available for this study.

### Vaccine Coverage and Macrolide Use

Because studies estimating vaccine coverage used different methods among countries, we used vaccine doses to estimate a proxy of vaccine coverage. Data on monthly vaccine doses sold or distributed were provided by the PCV7 manufacturer (Wyeth, Brussels, Belgium; Madrid, Spain; Maidenhead, UK; and Paris, France) and by the Health Protection Agency for doses distributed by the UK Department of Health. Assuming that all doses were administered to children <2 years of age at an average of 3 doses per child (allowing for missed doses and catch-up schedules), we calculated the proportion of children <2 years of age who should have received an average of 3 PCV7 doses. We also calculated the number of vaccine doses distributed per 1,000 children <5 years of age for comparison with serotype-specific incidences in children <5 years of age.

We collected data on use of antimicrobial drugs from the European Surveillance of Antimicrobial Consumption (*20*). We compared these data with the serotype-specific incidence of antimicrobial drug–resistant isolates.

### Data Analysis

For all incidence calculations, we adjusted numbers of cases to the rate of underreporting to the NRL to estimate total numbers of cases and control for surveillance enhancement over time. Underreporting rates were calculated by country, year, and age group by dividing the respective number of cases with an isolate typed at the NRL by the total number of laboratory-confirmed IPD cases estimated in the country. Total numbers of IPD cases were estimated by different methods: in Belgium and France, through periodic capture**–**recapture studies and correction for laboratory coverage (*7**,**21*); in England and Wales, by reconciliation of 2 large datasets (*22*); in Spain, by calculation of hospital underreporting rates as a proxy (*23*). Age-specific incidence rates were computed by dividing adjusted numbers of cases by the respective midperiod population of each country and age group, by using population figures from Eurostat for Spain and from the national institutes for statistics of Belgium, France, and England and Wales. For serotype-specific incidence calculations, cases with missing serotype data were accounted for by multiplying the overall incidence by the yearly proportions of serotype-specific disease in each age group (*12*).

Trends in incidence over time were tested for linear model by the *t* test, except for France because of missing data points; this model showed an overall better fit for the 3 studied serotypes. Correlation between vaccine doses per 1,000 children <5 years of age and serotype-specific incidence over years was tested by the Pearson correlation test, allowing for a 6-month lag between vaccine use and incidence. We also compared the annual average of serotype-specific incidence of the prevaccine period with the postmarketing period and computed incidence rate ratios (IRRs) and their exact 95% confidence intervals; p values were calculated by the Fisher exact test. We considered p values <0.05 significant. All statistical analyses were calculated by using STATA version 10.1 (StataCorp, College Station, TX, USA).

## Results

### PCV7 Coverage

**Figure 1:**
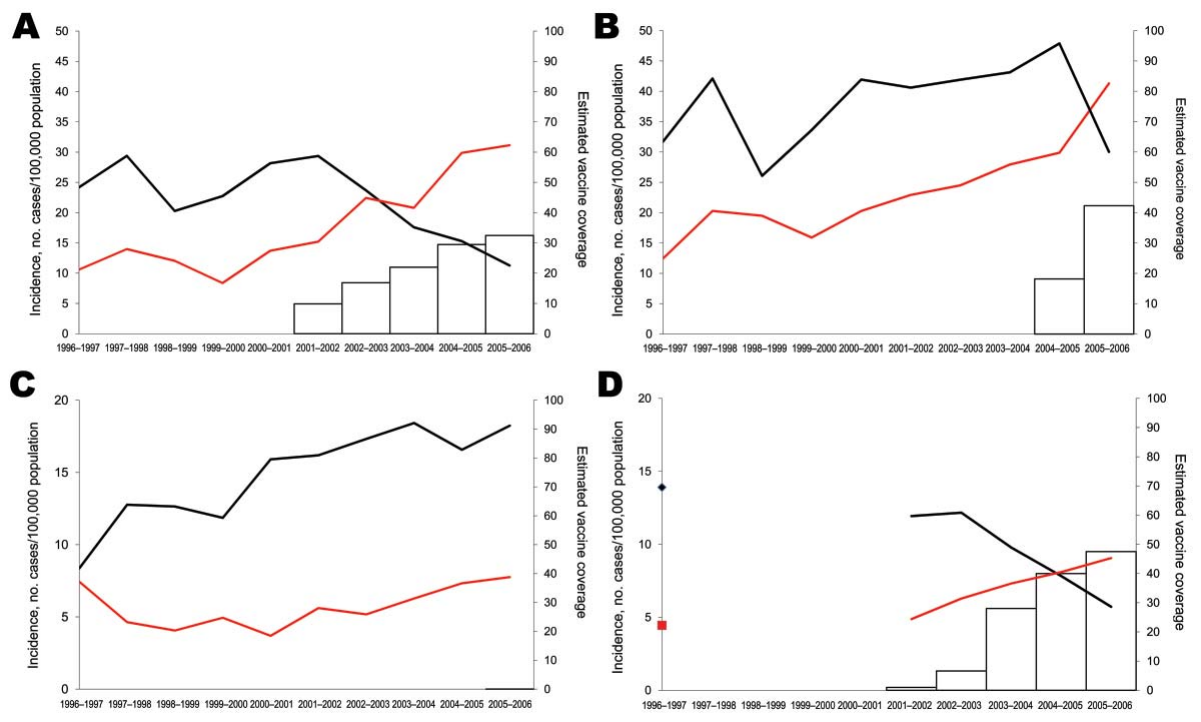
Incidence of pediatric invasive pneumococcal disease among children <5 years of age, by heptavalent pneumococcal conjugate vaccine (PCV7) (black lines) and non-PCV7 (red lines) serotypes, for A) Spain, B) Belgium, C) England and Wales, and D) France, 1996–2006. Estimated vaccine coverage is the annual number of PCV7 schedules per 100 children <2 years of age, assuming an average of 3 doses administered to each child. Vaccine coverage is not visible for England and Wales because it remains <1%.

PCV7 use began in Spain in 2000–2001, France in 2001–2002, Belgium in 2004–2005, and England and Wales in 2005–2006 and increased gradually in the first 3 countries (Figure 1). In 2005–2006, the proportion of children <2 years of age who had received an average of 3 PCV7 doses was ≈33% in Spain, ≈48% in France, ≈42% in Belgium, and <1% in England and Wales.

### Overall Incidence

During July 1996–June 2006, the NRLs of the 4 countries reported 13,584 IPD cases among children <15 years of age: 3,170 cases in Spain, 2,862 in Belgium, 2,188 in France, and 5,364 in England and Wales. Data were not available from France from 1997–98 through 2000–2001 because NRL activities stopped in 1997 and began again in 2001. The proportion of confirmed IPD cases typed at the NRLs increased during the study period because of improving reporting of each NRL (Table 1). IPD incidence per 100,000 children <15 years of age increased during the 10-year period in Spain, Belgium, and England and Wales from 14.0 to 18.5, 20.1 to 28.2, and 6.1 to 10.6, respectively, but remained fairly stable in France, ≈7.0.

**Table 1:** Cases of invasive pneumococcal disease, underreporting rates, and numbers adjusted for underreporting among children <15 years of age, 4 European countries, 1996–2006*

Country/epidemiologic year	Total typed	Underreporting, %†	Total adjusted‡	Serotype				
				PCV7 types	NVT	1	7F	19A
Spain								
1996–97	155	18	869	544	325	62	0	28
1997–98	179	19	919	590	329	41	15	5
1998–99	191	26	740	415	326	81	16	31
1999–00	211	30	713	463	250	88	7	20
2000–01	252	28	901	580	322	107	11	54
2001–02	301	30	1,014	613	401	91	3	44
2002–03	426	39	1,092	528	564	131	31	97
2003–04	434	45	959	433	526	106	22	128
2004–05	528	43	1,228	367	860	207	70	198
2005–06	493	42	1,185	288	896	312	74	168
Belgium								
1996–97	160	44	363	225	138	34	7	11
1997–98	202	46	436	272	165	32	9	32
1998–99	185	55	339	179	159	31	7	29
1999–00	197	55	356	218	138	40	5	23
2000–01	256	56	455	293	162	52	11	32
2001–02	295	65	454	268	186	48	11	32
2002–03	370	75	497	283	214	66	15	42
2003–04	382	73	521	270	251	91	20	42
2004–05	437	76	578	315	263	103	22	45
2005–06	378	75	506	190	316	102	37	58
France								
1996–97	258	33	773	547	226	52	0	40
1997–98	NA	NA	NA	NA	NA	NA	NA	NA
1998–99	NA	NA	NA	NA	NA	NA	NA	NA
1999–00	NA	NA	NA	NA	NA	NA	NA	NA
2000–01	NA	NA	NA	NA	NA	NA	NA	NA
2001–02	320	41	775	497	278	64	15	53
2002–03	399	46	864	531	333	92	32	87
2003–04	384	47	811	424	387	119	23	90
2004–05	438	54	806	359	447	159	41	71
2005–06	389	50	774	277	497	157	62	93
England and Wales								
1996–97	378	60	630	315	315	45	13	13
1997–98	429	62	689	462	226	43	14	26
1998–99	438	67	654	473	181	24	10	16
1999–00	429	64	666	453	213	36	8	37
2000–01	518	71	735	562	173	37	23	21
2001–02	557	72	770	549	221	37	14	28
2002–03	541	67	804	579	224	37	24	30
2003–04	627	71	889	628	261	67	16	37
2004–05	672	74	914	590	324	114	20	39
2005–06	775	76	1,014	640	374	139	38	38

*PCV7, heptavalent pneumococcal conjugate vaccine; PCV7 types, serotypes 4, 6B, 9V, 14, 18C, 19F, and 23F; NVT, non-PCV7 vaccine serotypes, i.e., serotypes not included in the PCV7 vaccine; NA, not available. †Underreporting rate = number of cases for which an isolate was typed at the national reference laboratory on the total number of laboratory-confirmed invasive pneumococcal cases estimated in the country (by capture–recapture or other methods) in children <15 years of age. ‡The sum of PCV7 types and NVT cases may slightly differ from the total adjusted because of rounding. §Data have been adjusted for ascertainment and for the distribution of blood/cerebrospinal fluid isolates.

Isolates from CSF represented 15% of invasive isolates in patients <15 years in Spain, 11% in Belgium, 16% in England and Wales, and 32% in France. Because blood isolates were underrepresented in France, NRL data were adjusted to the CSF/blood distribution reported by national epidemiologic surveillance, by year and age group (*7*). Only adjusted data are presented here.

### PCV7-Type and NVT IPD Cases

**Figure 2:**
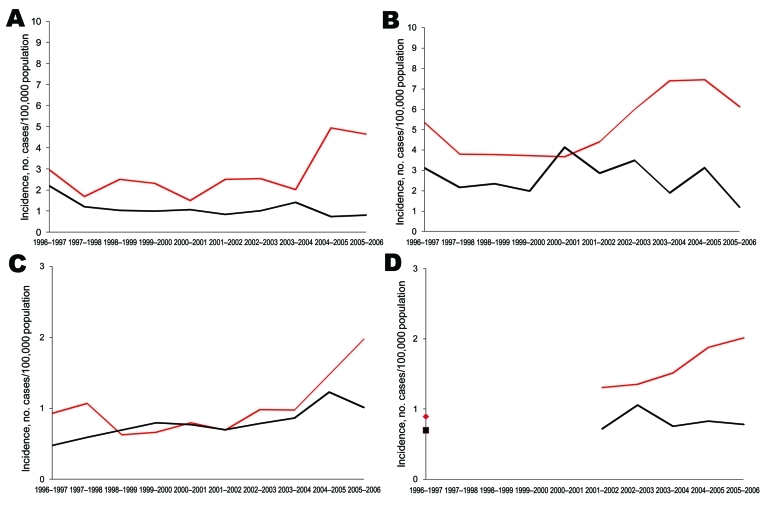
Incidence of pediatric invasive pneumococcal disease among children 5–14 years of age, by heptavalent pneumococcal conjugate vaccine (PCV7) (black lines) and non-PCV7 (red lines) serotypes, A) Spain, B) Belgium, C) England and Wales, and D) France, 1996–2006.

In children <5 years of age, incidence of PCV7-type disease started to decrease shortly after PCV7 introduction in Spain, Belgium, and France; the decrease was inversely related to increasing vaccine sales (Figure 1). Between the prevaccine period and the last study year (2005–2006), PCV7-type IPD significantly declined by 58%, 22%, and 52%, respectively, in these 3 countries (Table 2). In England and Wales, where vaccine sales were negligible, PCV7-type IPD increased by 25%, but the proportion of IPD caused by PCV7-type decreased slightly, from 75% to 70% (p = 0.004). In older children, PCV7-type IPD showed no clear trend, except in Belgium, where it significantly decreased (Figure 2; Table 3).

**Table 2:** Serotype-specific adjusted incidence rates of invasive pneumococcal disease in children <5 years of age before and after marketing of PCV7, Spain, Belgium, France, and England and Wales*

Country/serotypes	Incidence rate†		Incidence rate ratio (95% CI)	p value
	Prevaccine (1999–2002)	Postmarketing (2005–2006)		
Spain				
PCV7 types	26.8	11.3	0.4 (0.4–0.5)	<0.001
Non-PCV7 types	12.5	31.1	2.5 (2.2–2.8)	<0.001
1	2.7	8.1	3.0 (2.4– 3.7)	<0.001
7F	0.2	3.0	16.9 (8.6–37.7)	<0.001
19A	1.9	7.2	3.7 (2.9–4.8)	<0.001
Belgium				
PCV7 types	38.7	30.0	0.8 (0.7–0.9)	0.002
Non-PCV7 types	19.7	41.3	2.1 (1.8–2.5)	<0.001
1	3.4	9.5	2.8 (1.9– 4.1)	<0.001
7F	1.1	5.9	5.3 (2.9–9.8)	<0.001
19A	4.4	9.7	2.2 (1.5–3.1)	<0.001
France				
PCV7 types	11.9	5.7	0.5 (0.4–0.6)	<0.001
Non-PCV7 types	4.9	9.0	1.9 (1.5–2.2)	<0.001
1	0.6	1.5	2.7 (1.6–4.7)	<0.001
7F	0.3	1.3	4.2 (2.2–8.6)	<0.001
19A	1.3	2.4	1.9 (1.3–2.7)	<0.001
England and Wales				
PCV7 types	14.6	18.2	1.3 (1.1–1.4)	<0.001
Non-PCV7 types	4.7	7.8	1.6 (1.4–1.9)	<0.001
1	0.5	1.9	3.8 (2.6–5.8)	<0.001
7F	0.4	0.9	2.2 (1.3–3.7)	0.002
19A	0.8	1.0	1.3 (0.8–2.0)	0.271

*PCV7, heptavalent pneumococcal conjugate vaccine; CI, confidence interval. Marketing indicates that the vaccine was marketed and available for use in the country but not introduced in the vaccine schedule free of charge. Vaccine coverage differed by country during this period, ranging from 33% to 48% in Spain, Belgium, and France but <1% in England and Wales. PCV7 types include serotypes 4, 6B, 9V, 14, 18C, 19F, and 23F. †Cases per 100,000 children <5 years of age. Data for 1999–2002 are annual averages. Prevaccine period is 2001–2002 for France (data not available for previous years).

**Table 3:** Serotype-specific adjusted incidence rates of invasive pneumococcal disease in children 5–14 years of age before and after marketing of PCV7, Spain, Belgium, France, and England and Wales*

Country/serotype	Incidence rate†		Relative risk (95% CI)	p value
	Prevaccine (1999–2002)	Postmarketing (2005–2006)		
Spain				
PCV7 types	1.0	0.8	0.8 (0.6–1.3)	0.387
Non-PCV7 types	2.1	4.6	2.2 (1.8–2.7)	<0.001
1	1.1	3.1	2.9 (2.3–3.7)	<0.001
7F	0.1	0.2	2.0 (0.7–5.6)	0.175
19A	0.06	0.12	2.1 (0.5–7.7)	0.226
Belgium				
PCV7 types	3.0	1.2	0.4 (0.2–0.7)	<0.001
Non-PCV7 types	3.9	6.1	1.6 (1.2–2.1)	0.003
1	2.2	3.8	1.8 (1.2–2.6)	0.003
7F	0.2	0.3	1.2 (0.2–5.2)	0.728
19A	0.3	0.1	0.2 (0.0–1.4)	0.094
France				
PCV7 types	0.7	0.8	1.1 (0.7–1.6)	0.664
Non-PCV7 types	1.3	2.0	1.5 (1.2–2.0)	0.001
1	0.6	1.3	2.3 (1.6–3.4)	<0.001
7F	0.0	0.1	3.6 (1.0–20.2)	0.038
19A	0.1	0.0	0.3 (0.0–1.6)	0.116
England and Wales				
PCV7 types	0.7	1.0	1.4 (1.0–1.9)	0.026
Non-PCV7 types	0.7	2.0	2.9 (2.3–3.6)	<0.001
1	0.3	1.3	4.2 (3.0–5.9)	<0.001
7F	0.0	0.2	5.5 (1.9–18.0)	0.001
19A	0.05	0.1	2.1 (0.7–5.9)	0.148

*PCV7, heptavalent pneumococcal conjugate vaccine; CI, confidence interval. Marketing indicates that the vaccine was marketed and available for use in the country but not introduced in the vaccine schedule free of charge. Vaccine coverage differed by country during this period, ranging from 33% to 48% in Spain, Belgium, and France but <1% in England and Wales. PCV7 types include serotypes 4, 6B, 9V, 14, 18C, 19F, and 23F. †Cases per 100,000 children 5–14 years of age. Data for 1999–2002 are annual averages. Prevaccine period is 2001–2002 for France (data not available for previous years).

In contrast, incidences of NVT significantly increased in all 4 countries during the 10-year period (p<0.001), mainly from 2000–2002 onward (Figures 1, 2). From the prevaccine period to 2005–2006, NVT IPD increased significantly in both the <5-year and the 5**–**14-year age groups in all countries (Tables 2, 3). In each country, NVT IPD in the <5-year age group began to increase before PCV7 was introduced and gradually increased from year to year during 2002**–**2006. In children 5**–**14 years of age, NVT IPD fluctuated during the study period but mostly increased during 2002**–**2006. Serotypes 1, 7F, and 19A contributed most to this increase, representing 61% (range 57%**–**63%) of NVT IPD in children <15 years of age in 2005–2006. The dynamics of these 3 serotypes differed in terms of time trends and age groups affected.

#### Serotype 1

**Figure 3:**
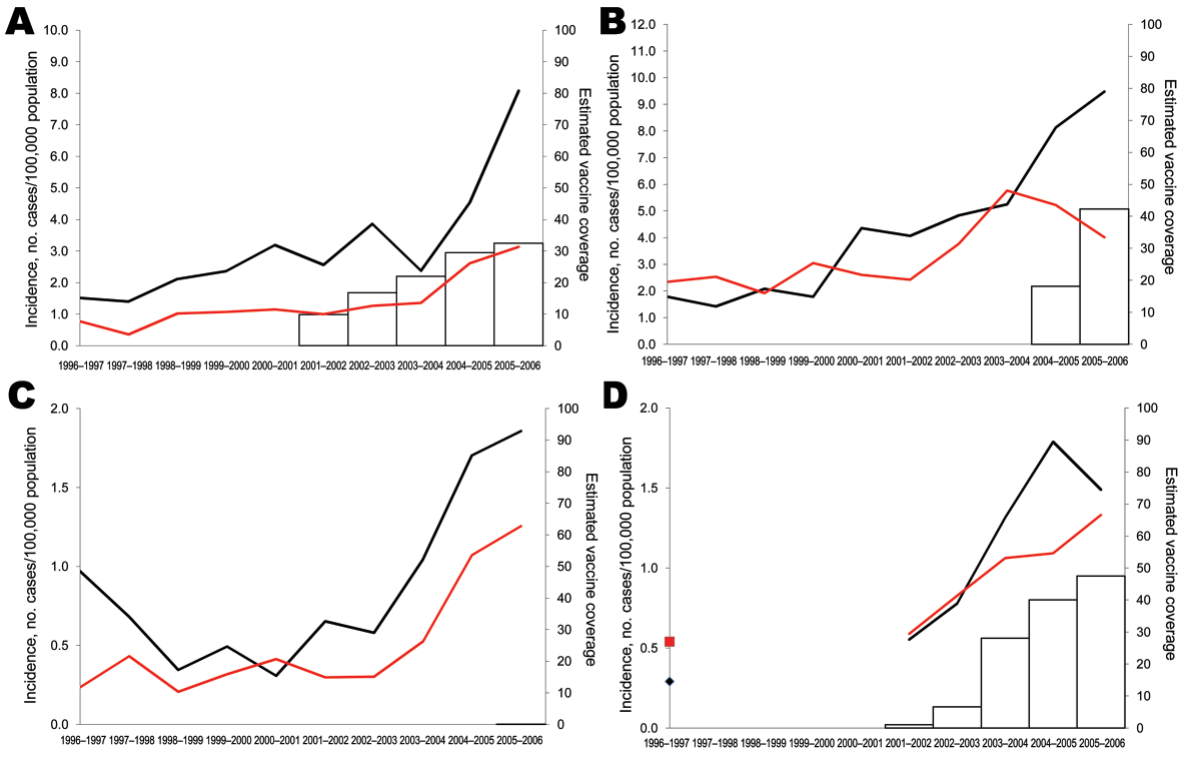
Incidence of invasive pneumococcal disease in children caused by serotype 1 for children <5 years of age (black lines) and 5–14 years of age (red lines), in A) Spain, B) Belgium, C) England and Wales, and D) France, 1996–2006. Estimated vaccine coverage is the annual number of PCV7 schedules per 100 children <2 years of age, assuming an average of 3 doses administered to each child. Vaccine coverage is not visible for England and Wales because it remains <1%.

Serotype 1 disease increased significantly in each country during the 10-year period in both age groups (Figure 3). In children <5 years of age, the increase in serotype 1 began before PCV7 sales began. However, the largest increases occurred from 1999**–**2002 to 2005–2006, when incidence increased by 2**–**4-fold in both age groups in all countries (Tables 2, Table 3). Increases in the incidence of serotype 1 did not correlate significantly with PCV7 sales, except in France (Pearson r = 0.903, p = 0.036). In the 5**–**14-year age group, incidences were lower, but in 2005–2006, serotype 1 constituted in average 50% of IPD in that age group compared with 13% in children <5 years of age. All serotype 1 isolates were susceptible to penicillin. Incidence and proportion of erythromycin-resistant serotype 1 was low but increased in Belgium in 2004–2006.

#### Serotype 7F

**Figure 4:**
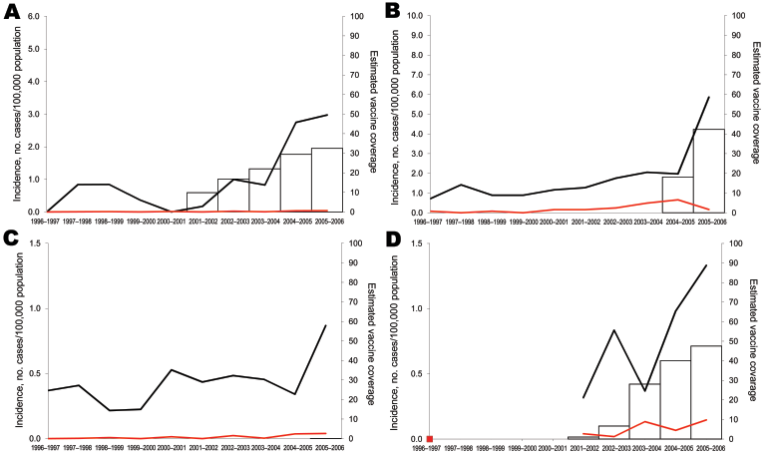
Incidence of invasive pneumococcal disease in children caused by serotype 7F for children <5 years of age (black lines) and 5–14 years of age (red lines), in A) Spain, B) Belgium, C) England and Wales, and D) France, 1996–2006. Estimated vaccine coverage is the annual number of PCV7 schedules per 100 children <2 years of age, assuming an average of 3 doses administered to each child. Vaccine coverage is not visible for England and Wales because it remains <1%.

Most (74%) serotype 7F cases occurred among children <5 years of age. In this group, IPD increased substantially in each country, mostly during 2004**–**2006, and correlated significantly with PCV7 sales, except in France (Pearson r = 0.901, p = 0.037 in Spain; r = 0.988, p = 0.002 in Belgium; r = 0.965, p = 0.008 in England and Wales; and r = 0.746, p = 0.148 in France) (Figure 4). From the prevaccine period to 2005–2006, incidence increased most in Spain and least in England and Wales (Table 2). In children 5**–**14 years of age, incidence rates also increased (Table 3), but numbers of cases were small. All serotype 7F isolates were susceptible to penicillin, and only 6/315 isolates in children <5 years of age were erythromycin resistant.

#### Serotype 19A

**Figure 5:**
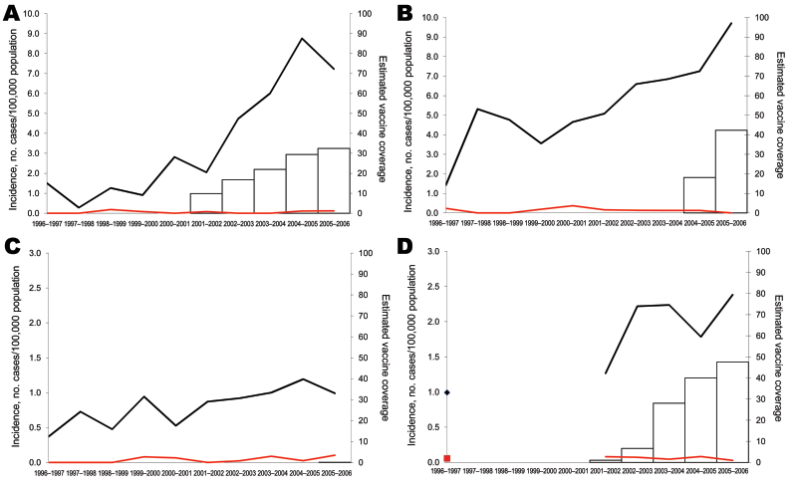
Incidence of invasive pneumococcal disease in children caused by serotype 19A for children <5 years of age (black lines) and 5–14 years of age (red lines), in A) Spain, B) Belgium, C) England and Wales, and D) France, 1996–2006. Estimated vaccine coverage is the annual number of PCV7 schedules per 100 children <2 years of age, assuming an average of 3 doses administered to each child. Vaccine coverage is not visible for England and Wales because it remains <1%.

Serotype 19A disease affected predominantly children <5 years of age (94% of cases), for whom incidence rates more than doubled (IRR range 2.4**–**6.8) over the period (Figure 5). Incidence had already increased before PCV7 sales started in Belgium, Spain, and England and Wales, but the trend was significant only in Belgium (during 1996**–**2004). After PCV7 marketing, increases correlated significantly with vaccine sales in Spain and Belgium (Pearson r = 0.929 and 0.884, p = 0.022 and 0.047, respectively). From the prevaccine period to 2005–2006, 19A incidence significantly increased in Spain, France, and Belgium (Table 2); in England and Wales, the 27% increase was not significant. In children 5–14 years of age, numbers of cases were too small to identify any significant change.

**Figure 6:**
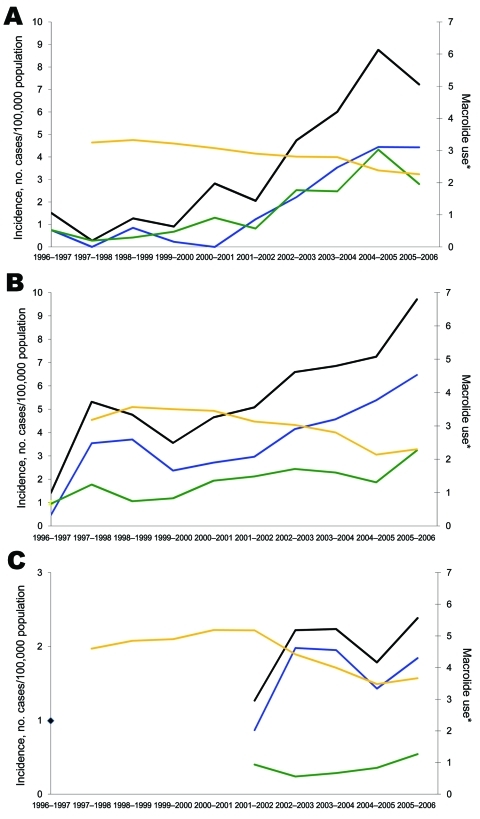
Incidence of serotype 19A invasive pneumococcal disease in children <5 years of age (black lines) showing breakdown of erythromycin-resistant (blue lines) versus -susceptible (green lines) infections and rate of macrolide use (gold line) in outpatient settings for A) Spain, B) Belgium, and C) France, 1996–2006. *Defined daily doses per 1,000 inhabitants per day.

Serotype 19A isolates showed high and increasing levels of antimicrobial drug resistance in Belgium, Spain, and particularly in France (data unavailable from England and Wales). In the <5-year age group, the prevalence of nonsusceptible strains ranged 0%–50% in 1996–1997 and increased in 2005–2006 to 21%, 48%, and 86% for penicillin and 67%, 61%, and 77% for macrolides in Belgium, Spain, and France, respectively. Full penicillin resistance was rare (0%**–**6% of isolates). Spain and Belgium shared similar patterns: incidence of penicillin-susceptible 19A increased more than resistant strains; incidence of erythromycin-resistant strains increased more than erythromycin-susceptible strains (Figure 6); in 1998**–**2006, the use of penicillin in ambulatory setting, calculated in defined daily doses per 1,000 inhabitants per day, increased slightly, and macrolide use declined by 31%**–**38%. In France, where nonsusceptible isolates predominated, use of penicillin and macrolides initially was much higher than that in the other countries but declined (**–**17% and **–**37%, respectively) until 2006. In England and Wales where use of antimicrobial drugs was initially lower, macrolide use became similar to Belgium and Spain from 2004 onward, but data on resistance were not available.

### Evolution of Meningitis

Numbers of meningitis cases caused by individual serotypes were small, especially for serotype 1. In children <5 years of age, the incidence of meningitis from serotypes 19A and 7F combined also increased significantly in Spain, Belgium, and France from the prevaccine period to 2005–06 (IRR 3.8, 4.9 and 2.3, respectively, p<0.001); in England and Wales, it did not increase significantly (IRR 1.3, p = 0.283).

## Discussion

This study compared the dynamics of NVT disease in 4 countries before universal PCV7 vaccination. In Spain, Belgium and France, serotype 1, 7F and 19A incidence increased considerably under rising (though moderate) vaccine coverage. In England and Wales, where PCV7 use was negligible, serotype 1 disease increased substantially, 7F disease rose less than in the other countries, and 19A disease increased nonsignificantly. The proportion of IPD caused by serotype 19A remained stable in England and Wales, and the incidence of meningitis caused by 19A and 7F did not change significantly, suggesting that an increase in case detection caused part of the changes in 19A and 7F incidence. Indeed, a study showed that the 21% increase in IPD incidence in southwest England during 1996**–**2005 was no longer observed after adjustment for annual blood-culturing rates (*24*).

In Spain, the marked increase in NVT occurred concomitantly with PCV7 use, which led several studies to conclude that vaccine-induced replacement of serotypes had largely contributed to this increase (*6**,**11*). On the basis of our study findings, we suggest that vaccine use contributed to the increase in serotypes 7F and 19A. Both serotypes increased markedly under increasing PCV7 coverage and remained stable or increased less in the countries not using PCV7. Increases in 19A and 7F incidences correlated significantly with increasing vaccine sales in Spain and Belgium and were more pronounced in children <5 years of age, at whom PCV7 was aimed, than in older children. In Belgium and Spain, where both serotypes had already increased before PCV7 introduction, its slope escalated after PCV7 use. Although these data were observed under moderate PCV7 coverage, recent data from Belgium, England and Wales, and France indicate additional increases in the adjusted incidences of serotypes 7F and 19A under universal vaccination (2007–08) and high vaccine coverage (*25**–**27*).

However, we also suggest that vaccine-induced serotype replacement alone cannot explain the increase in NVT. First, serotype 1 rose well before PCV7 marketing in Belgium and Spain, affected predominantly older age groups, and increased in England and Wales in the absence of vaccine use. Second, serotype 19A increased in Belgium and Spain before PCV7 use. Similarly, serotype 7F or 19A disease also increased in countries not using PCV7 (*28**–**30*). Third, some EU countries with widespread PCV7 use did not experience similar rises (*31**,**32*).

Other factors most likely contributed to the increases. Cyclical trends of serotype 1 were described in Scandinavian countries before any PCV7 use (*3**,**33**,**34*). A wave of serotype 1 (and possibly 7F) may have occurred in these 4 neighboring countries. Conversely, the high use of antimicrobial drugs, especially macrolides, allegedly favored the increase of nonsusceptible serotype 19A (*14**,**35*). A modeling study suggested that use of antimicrobial drugs played a larger role than did PCV7 use in the increase of resistant 19A in the United States (*36*). In 3 countries in our study where 19A incidence (and resistant strains) increased, use of antimicrobial drugs was higher than in England and Wales where 19A stayed stable. However, incidence of penicillin- and erythromycin-susceptible 19A strains also increased in Spain and Belgium. Macrolide use decreased 37%**–**41% during 1998**–**2006 in the 3 countries, whereas 19A incidence increased 80%**–**253%. Similarly, serotype 19A incidence increased in England and Wales after universal PCV7 vaccination despite stable macrolide use (*26*). The role of antimicrobial drugs is thus difficult to delineate and suggests a synergistic effect of antimicrobial drugs and PCV7. Other factors for replacement have been raised (high prevalence of NVT carriage and low vaccine coverage), but they conflict with current knowledge (*37*): 7F is a rarely carried serotype, and 19A and 7F increased further under higher PCV7 coverage. These conflicting views suggest that factors leading to replacement disease are still not fully understood; its cause is probably multifactorial and population dependant.

Our study has several limitations. First, enhancement of pediatric IPD surveillance and possible changes in blood culture practices could not be completely controlled by our methods of adjusting for underreporting. This limitation certainly applied to England and Wales, where reconciliation of 2 datasets could not totally adjust for the increase in case reporting and blood culturing, which most likely contributed to the increased incidence in nearly all serotypes studied, probably leading to overestimation of the NVT increase in England and Wales. Increase in blood culturing over time in the other countries is not suggested by the sharp decrease in PCV7-type IPD, the similar trends in serotype-specific meningitis incidence (based on CSF isolation), and data on blood cultures in hospitals in Belgium (+13% from 1999**–**2002 to 2005–2006 while NVT IPD increased 210%). Second, missing serotype data (more frequent in the prevaccine period) may have led to imprecision in serotype distributions; however, similar age-specific PCV7-type and NVT distributions and trends were observed in other studies in Belgium, France, Spain, and England (*5**–**8**,**24*), PCV7 serotype coverage did not vary with the geographic origin of pneumococcal strains in France (*7*), and the age and sample distribution of children for whom serotype information was available did not differ from that of other children in the Belgium dataset. Finally, estimation of vaccine coverage assumed that all PCV7 doses were administered at an average schedule of 3 doses for children <2 years of age. This method may overestimate PCV7 coverage because a proportion of children are likely to be incompletely vaccinated given the high cost of PCV7 paid for by parents (*38*), but it may also underestimate coverage because many children received fewer doses in catch-up vaccination. However, coverage values were close to those estimated by population surveys (*6**,**7**,**11**,**38*).

Such an ecologic study cannot determine which rise in disease incidence is attributable to vaccine, secular trends, or use of antimicrobial drugs, and other possible factors may have contributed. However, the strength of this study is in the comparison of epidemiologic changes in 4 countries showing variations in serotype dynamics, vaccine use, and antimicrobial drug use.

The increase in incidence of serotypes 1, 7F, and 19A has partly countered the positive impact of PCV7 on overall IPD incidence in the first 2**–**5 years of nonuniversal vaccine use in Belgium, France, and Spain. The new 10-valent (1 and 7F) or 13-valent (1, 7F, and 19A) conjugate vaccines include these serotypes. However, a better understanding of serotype dynamics and contribution of vaccine and antimicrobial drug use is essential to guide decisions on the implementation of new vaccines and to assess their impact. Multicountry studies are useful for comparing serotype dynamics among population groups that have different levels of vaccine and antimicrobial drug use, but analyses should account for underreporting and prevaccine trends.
